# Basalt/Glass Fiber Polypropylene Hybrid Composites: Mechanical Properties at Different Temperatures and under Cyclic Loading and Micromechanical Modelling

**DOI:** 10.3390/ma14195574

**Published:** 2021-09-25

**Authors:** Anna Kufel, Slawomir Para, Stanisław Kuciel

**Affiliations:** 1 Faculty of Materials Engineering and Physics, Institute of Materials Engineering, Cracow University of Technology, Warszawska 24, 31-155 Cracow, Poland; 2 Faculty of Mechanical Engineering, Institute of Automotive Engineering and Internal Combustion Engines, Cracow University of Technology, Warszawska 24, 31-155 Cracow, Poland; s.para@sbg.at

**Keywords:** hybrid composite, basalt fiber, glass fiber, hysteresis loops, mechanical properties, FEA

## Abstract

Basalt/glass fiber polypropylene hybrid composites were developed as subjects of investigation, with the aim to characterize their properties. An injection molding machine was used to produce the test samples. The following three different tests, at various specimen temperatures, were conducted: tensile test, three-point flexural test, and Charpy impact test. To determine fatigue behavior, the samples were uniaxially loaded and unloaded. Mechanical hysteresis loops were recorded and the dissipation energy of each loop was calculated. To determine the adhesion and dispersion between the fibers and the matrix, the fractured surfaces of the various specimens, after the tensile test, were investigated using a scanning electron microscope. The results show that the production of a composite with both basalt and glass fibers, in a polypropylene matrix with maleic anhydride-grafted polypropylene, can be successfully achieved. The addition of the two types of fibers increased the tensile strength by 306% and the tensile modulus by 333% for a composition, with 20% by weight, of fibers. The material properties were estimated with the help of a simulation software, and validated with a FEA. A satisfactory correlation between the simulation and measurement data was achieved. The error lays in a range of 2% between the maximum stress values. At a lower strain (up to 0.02), the stress values are very well matched.

## 1. Introduction

Glass fiber-reinforced composites are commonly used around the world. These materials are suitable for the following industries: automotive—door panels, engine cover, bumpers; marine—boat construction; medical—X-ray beds; aerospace—engine cowlings, seating, cabin interior parts; as well in home applications—windows, roof sheets, tables [1]; and they have applications in civil engineering, as presented in [2]. The most popular E-glass fibers are characterized by the following mechanical properties: a tensile strength of 2700–3000 MPa, elastic modulus of 72–76 GPa, and a maximum application temperature of 380 °C. Much research studied composites reinforced by glass fibers [3,4,5,6].

Basalt fibers can be a great alternative to glass fibers, as other scientists have shown [7,8]. Basalt is considered as a natural material, and it is produced from volcanic rock. The major advantage of basalt is that it is an environmentally friendly material, is non-toxic, and is non-carcinogenic. Compared to glass fibers, it has good mechanical properties, better thermal resistance, and higher chemical stability. The tensile strength of basalt fibers is 3000–3400 MPa, and they have an elastic modulus of 86–90 GPa and a maximum application temperature of around 600 °C. The production of basalt and glass fibers is similar; however, to produce basalt fibers, no additives are required, as in the case of glass fibers. The disadvantage of basalt fibers is the higher density and price. The applications of basalt fiber composites are, for example, the following: fire-proof doors, interiors, and sound isolation material in buildings. They are also used in road construction, the concrete industry, and in civil engineering applications, because of their high fire resistance. The interest of basalt increasingly comes into the focus of several publications [9,10,11].

It is of great interest to find materials with new properties. Hybridization is a good option to overcome some drawbacks. The combination of two kinds of fillers in a single matrix can show advantages of both fillers. The following different combinations of fillers can be used to produce hybrid composites: banana/glass [12], kenaf/glass [13], wood/glass [14], palm/kenaf [15], and biocarbon/basalt [16]. Hybridization can help to change the properties of different composite applications. Some of the benefits of hybridization are reduced costs of production, enhanced mechanical properties, better chemical resistance, and higher thermal stability. However, there is a need to achieve a balance of composite properties. Xian et al. [17] showed that, on the one hand, the combination of glass and carbon fibers can reduce the price of carbon fibers and, on the other hand, it can contribute to the improvement of the mechanical properties and poor fatigue resistance of glass fibers, by adding carbon fibers with excellent properties. The addition of glass fibers can maintain a cost reduction in composites when other, more expensive, fibers, such as basalt, are used [18]. Replacing some parts of glass fibers with carbon or basalt fibers may increase the resistance of the material to chemical corrosion. Article [19] studied the chemical resistance of carbon, glass, and basalt fibers. The authors used the following different solutions: water, acid, saline, and alkaline. Carbon fibers had the highest resistance to corrosive environments, followed by basalt fibers, and the lowest was E-glass fibers. After a treatment in acid and alkaline solution, E-glass fibers showed an extensive decrease in weight, because fibers contain boron, which is susceptible to chemical corrosion. The authors of [20] investigated hybrid composites with glass and basalt fibers. The hybridization improved the tensile and flexural behavior as well. Four different reinforcing fibers, within a PP as a matrix material, were used for mechanical investigations in [21]. Further, 30 wt.% of various compositions of fiber types were used as reinforcing materials. Additionally, hybrid compositions of two matrix materials and two or more reinforcing or filling materials were researched. In contrast to the actual article, the authors used a compression molding manufacturing method of the specimens. Saleem et al. [22] studied the effect of the fiber coating of basalt fiber hybrid composites on mechanical properties. As a second reinforcement, kenaf and flax, as bast fibers were used in the research. In article [23], the authors described the role of a coupling agent on the mechanical properties of cellulose/basalt polypropylene composites.

An additional aspect, and an overview of the utilization of different hybrid polymer composites in automotive applications, are presented by the authors of article in [24]. Hybridization of natural fibers with glass, carbon, or basalt enhanced the mechanical properties. To list some injection molded glass-reinforced polypropylene parts used within the automotive sector, according to Lutsey [25], interior parts of the vehicle, e.g., the console and shifter, are produced. Additional requirements of these parts, especially in the automotive sector, and the desired properties within the automotive industry, are presented by Volpe et al. in [26].

Nowadays, newly produced elements are designed and also re-designed by the help of CAx. To evaluate if the components will sustain the operating loads, and to enable a fast prediction to be made, finite element analyses (FEA) can be carried out, often by defining homogenous materials. If injection molded materials will be used, which consist of several components, advanced calculation methods for heterogenic material property computing will be needed. In this article, the polypropylene composite consists of two inclusions. This leads to the task of describing the mechanical properties of the matrix, and the basalt and glass fibers, which are orientated in a certain way. The fiber orientation is defined by the production method, and it has a huge impact on the anisotropic property of the composite. The inhomogeneity of composites requires micromechanical modelling, which can be realized by the help of programs such as Digimat. The method, therefore, is called ‘mean-field homogenization’ [27]. Several publications show the process of the mean-field homogenization of different materials, with just one inclusion, but the micromechanical modelling of the presented composite, based on polypropylene reinforced with basalt and glass fibers, is rarely reported in the literature. The following literature shows that a lot of research is carried out in the area of nonlinear calculations, which is validated with experiments. In [28], the authors show the process of numerical model verification, by making use of different solution alternatives. In [29], a polyamide composite with 30 wt.% glass fibers was studied. The authors conclude that the orientation of the fibers, as well as the mesh resolution, have an important effect on the results. The authors of [30] calculated a polypropylene specimen with 40 wt.% glass fibers, using a similar simulation approach, and, again, the main conclusion is that the correct fiber orientation within the model is critical to obtain accurate predictions. Another article [31] shows the importance of correct fiber orientation for the simulation process. The research was correlated with measurements. The study in [32] used a similar simulation approach for a polyamide composite with 50 wt.% glass fibers, and the obtained maximum relative error of the double inclusion homogenization was less than 9.7%. An interesting approach of the mean-field homogenization of a polypropylene matrix with glass fiber inclusions, built on SEM observations, allowed the mechanical behavior of the material to be predicted, and is presented in [33]. Another environmentally friendly polypropylene composite modelling approach (besides the composite proposed in this article) and variation with 10, 20, 30, 40, and 50 wt.% bamboo is presented in [34].

The fatigue properties of materials can be determined by carrying out long-time tests. This is realized by loading and unloading a specimen with defined forces at specified frequencies. The deterioration of the test specimen increases with each load cycle, normally up to total failure. To carry out the fatigue tests, there are several methods. The authors of [35] used the method of Lehr to investigate the fatigue behavior of biodegradable composites with flax fibers. To obtain the well-known S–N curves, the authors of [36] proposed an approach of fatigue investigation at three maximum stress levels and a stress ratio of 0.4, at two different frequencies (2 Hz and 4 Hz), to determine the fatigue performance of a carbon/glass hybrid rod. An interface shear experiment was carried out and compared to the developed 2D FE analysis.

This study aimed to investigate the effect on the mechanical properties of the following two fillers in the composite: basalt fibers and glass fibers. The thermal impact on these properties was also investigated. An FEA (finite element analysis) was carried out, in addition to the presented results. A simulation step gives information about the probable orientation of the filler inside the matrix, due to the molding process. Mean-field homogenization of the composite defines the overall mechanical properties. The calculated mechanical properties and the predicted orientation are the input, to simulate the sigma-epsilon characteristics of the composite specimen. To predict the low cycle fatigue behavior of the composites, loading–unloading forces on the manufactured specimens were applied and the dissipation energy was calculated. Micrographs made by scanning electron microscopy exhibit the characterization of the fiber–matrix interface of the two-fiber composite. 

The research about basalt/glass hybrid composites with a thermoplastic matrix are not well studied and published in the literature. Combining basalt and glass fibers inside one matrix is more popular for thermoset composites [37,38,39]. Moreover, the hybridization of glass fibers with other fibers than basalt has been studied more extensively [40,41,42]. Basalt fibers were added to produce more eco-friendly composites, to take advantage of its higher mechanical properties, and thermal and chemical resistance.

## 2. Materials and Methods

### 2.1. Materials

To produce hybrid composites the following materials were used:

1. Polypropylene Moplen HP 500N (Basell Orlen Polyolefins, Płock, Poland).
2. Basalt fibers (BCS17-6.4-KV16): nominal cutting length—6.4 mm, nominal diameter—17 μm (Basaltex, Wevelgem, Belgium).
3. Glass fibers (Krosglass ER 5001): nominal cutting length—6 mm, nominal diameter —10 µm (Krosglass, Krosno, Poland).
4. Coupling agent: anhydride maleic PP SCONA TPPP 9112 GA (MAPP) (Byk, Altana AG, Wesel, Germany).

Composites were produced in the Laboratory of Plastics Technology (Grupa Azoty SA, Tarnów, Poland). Engel ES 200/40 HSL injection molding machine (ENGEL GmbH, Schwertberg, Austria) was used to produce dumbbell-shaped specimens type A. Preliminary granulates of polymer and fibers were compounded by making use of a two-screw extruder and a gravimetric screw feeder. The injection speed was 60–90 mm/s, temperature in the zones 180 °C–220 °C, mold temperature 40 °C and the screw speed was 40 rpm. 

### 2.2. Methods of Testing

Hydrostatic method was used to measure the density (PN-EN ISO 1183) of the composites using an analytical balance RADWAG WAS 22W (Radwag, Radom, Poland). In this method, as a solvent ethanol was used.

The tensile (PN-EN ISO 527-1:2012) and three-point flexural test (PN-EN ISO 178:2011) were carried out using an MTS Criterion Model 43 universal testing machine (MTS Systems Corp., Eden Prairie, MN, USA). Maximum load was up to 30 kN. To measure accurate displacement and calculate the tensile modulus an MTS 634.31F axial extensometer was used. The traverse speed of the test was 5 mm/min. Impact strength was evaluated by Charpy impact test (PN-EN ISO 179-2) using the Zwick/Roell MTS SP (Zwick Roell Group, Ulm, Germany) testing machine. Unnotched samples were used. Additionally, the mechanical tests were performed at −24 °C, 23 °C, and 80 °C. At least 5 samples were tested for each test and the standard deviation was calculated.

The measured mechanical properties, which were obtained by using the aforementioned equipment, were used for the numerical computation. By a micromechanical modelling procedure these properties can be estimated and used for FE calculation of new designed parts. The following programs were utilized to simulate the composite material properties:

- Moldex3D—simulation of the injection molding process to generate the fiber orientation within the specimen (manufacturing data);
- Digimat-MF—reverse engineering of the material properties to set up material definition (Digimat material);
- Ansys—setup of a FAE model with defined mesh, boundary and load conditions (structural model) and post-processing of the results;
- Digimat-RP—numerical load analysis of the composite specimen by considering the fiber orientation and material data.

The material parameter estimation was carried out by comparing the Digimat-MF output curve to the measurement data of the PP10B10G composite (template). The estimated parameters are presented in Table 1.

As additional experiment, the composites were subjected to short-time fatigue tests. The tests were carried out in the uniaxial load–unload mode, with a speed of 5 mm/min using a Shimadzu AGS-X machine (Schimadzu, Kioto, Japan). The value of the minimum load was 200 N and the maximum load value was 1000 N. The load values were selected on the basis of the tensile test results. Mechanical hysteresis loops were recorded during the 50 load–unload cycles of the sample. These measurement data were taken as input to calculate the dissipation energy of each loop.

Scanning electron microscope (SEM) JEOL JSN5510LV (JEOL Ltd., Tokyo, Japan) was used to observe the microstructure of the samples after tensile test. Cressington 108 auto sputter coater (Cressington Scientific Instruments, Watford, UK) was used to coat the samples with gold.

## 3. Results and Discussion

### 3.1. Physic-Mechanical Characterization

Table 2 presents the composition content of the composites and the results of the density measurement. Different ratios were used to produce composites with a step-wise increasing content of the fillers: 10, 15, up to 20 wt.%. To enhance the adhesion between the fibers and the matrix, a coupling agent was used, in the quantity of 3% by weight, for all the compositions. The mass fraction ratio of the coupling agent was chosen according to the supplier’s recommendation and other research [43,44]. The addition of glass and basalt fibers caused a slight increase in the density of the materials. The addition of up to 20%, by weight, of fibers allowed lightweight composites, with good mechanical properties, to be produced.

**Figure 1:**
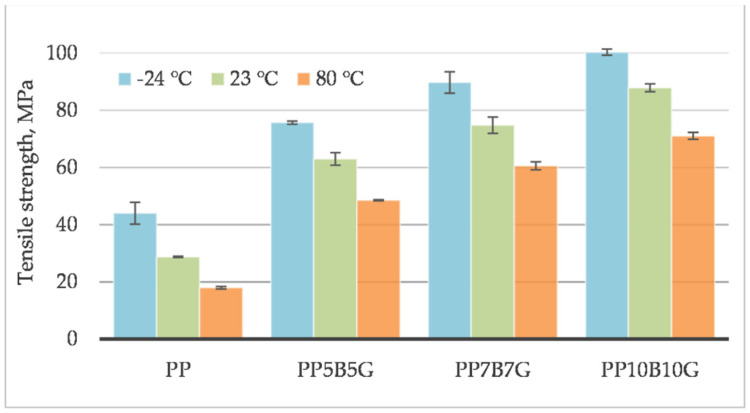
Tensile strength of basalt/glass fiber polypropylene composites at −24 °C, 23 °C, and 80 °C.

**Figure 2:**
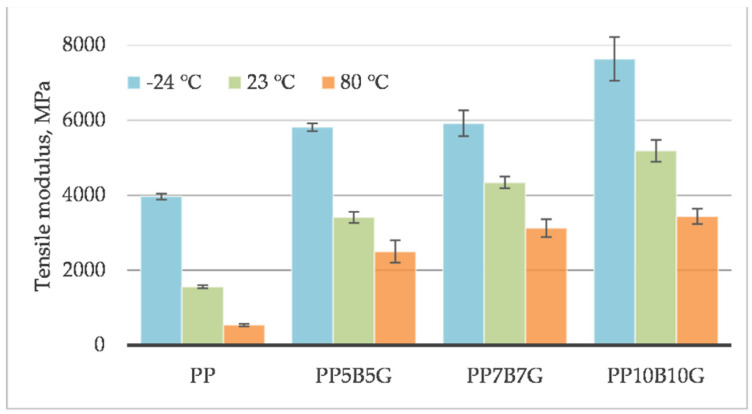
Tensile modulus of basalt/glass fiber polypropylene composites at −24 °C, 23 °C, and 80 °C.

**Figure 3:**
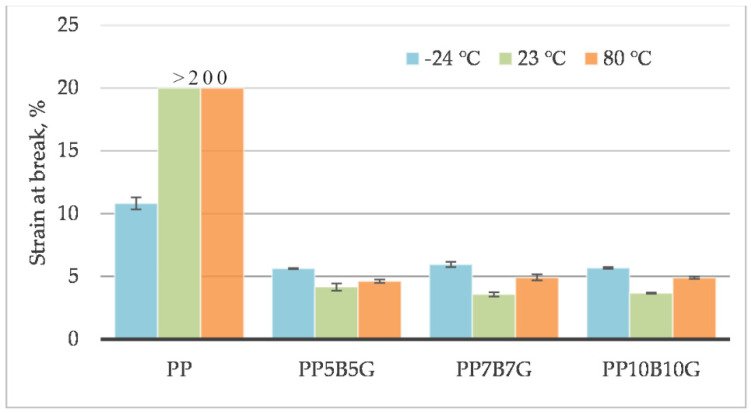
Strain at break of basalt/glass fiber polypropylene composites at −24 °C, 23 °C, and 80 °C.

The behavior of the material was measured at different operating temperatures. Tensile testing was conducted on specimens at 80 °C, 23 °C, and −24 °C. The tensile strength (Figure 1), tensile modulus (Figure 2), and strain at break (Figure 3) of the composites, which contained basalt fibers and glass fibers, were compared. For composites with 10%, by weight, of fibers, the tensile strength and tensile modulus increased by 119% and 219%, respectively. Further increasing the amount of filler (up to 20% by weight) resulted in an increase by 306% and 333%, respectively. At 80 °C, the composites showed a pseudo-ductile effect. With an increasing temperature, the tensile strength and elastic modulus values were found to be decreased for all the composites. This may be caused by thermal softening of the matrix. At lower temperatures, the material became more brittle. This is due to the glass transition temperature (T_g_) at which the behavior of the material changes. The predicted T_g_ of polypropylene homopolymer is −10 °C, but the actual value may be different, and depends on the heating rate and frequency [45]. The T_g_ and degree of crystallinity can be measured by differential scanning calorimetry (DSC). The addition of stiff fibers caused a decrease in the strain at break for all the compositions. The movability of the polymer chains decreased after encountering the obstacles—basalt and glass fibers. Further investigations are needed to know the results with a higher fiber content. In this study, focus was laid on low-cost and lightweight composites, reinforced with up to 20 wt.% fillers. Adding more fibers may result in an increase in mechanical properties and in a reduction in elongation.

**Figure 4:**
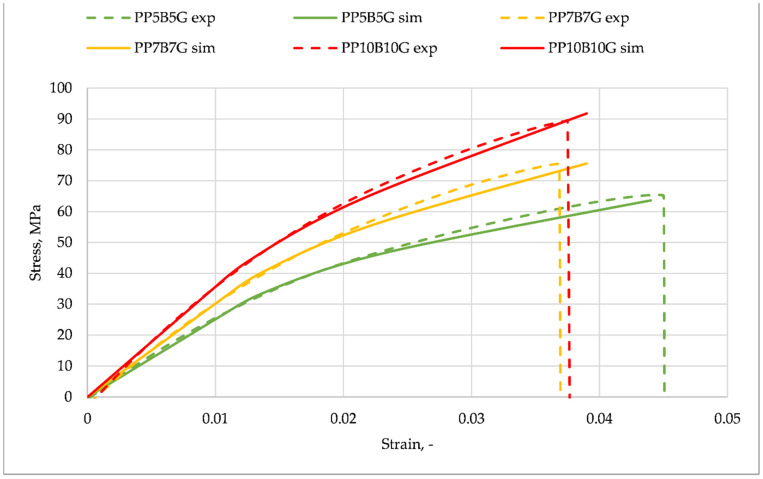
Stress–strain curves. Results of Digimat material analysis compared to measured data.

**Figure 5:**
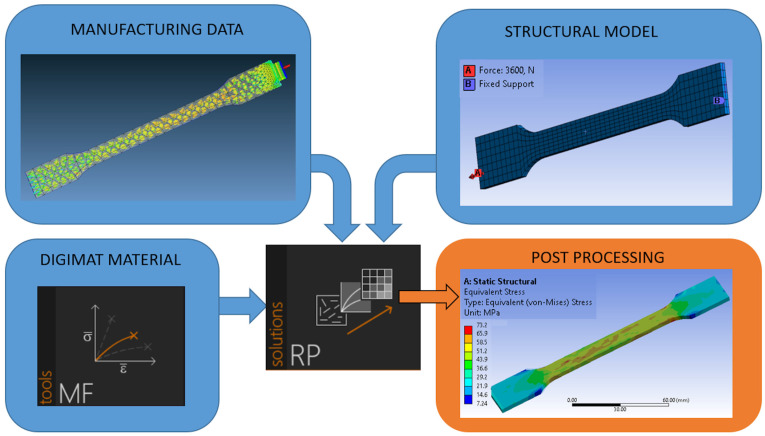
Computing flow chart.

With the aim to reproduce the real mechanical properties of the composites within an FEA study, the simulation program Digimat was used. A reverse engineering process allowed the program to be parametrized. Therefore, the real tensile test values served as a reference and were compared to the generated results. In contrast to other studies [46,47], which analyzed two-phase composites, the materials within this paper are described by multi-phase homogenization. Two-phase composites are made of a matrix material, which is reinforced with a certain number of identical inclusions, all having the same shape, material, and orientation. In this study, the number of inclusions was two (glass fibers and basalt fibers), and they have different mechanical properties, geometries, and densities. In Figure 4 the results of the homogenized material properties (presented as Digimat material in Figure 5, which shows the computing flow chart) were compared to the measurement data. The Digimat-MF material properties were gained by reverse engineering, based on the PP10B10G experimental data. A good correlation between the measurement and the simulation was achieved. The material properties for the other two fiber compositions were generated with Digimat-MF, by setting the appropriate phase (mass) fractions within the program. A small difference between the simulation and measurement data can be realized at strains up to a value of 0.02. The strictly calculated material data (7.5% and 5% by weight) show a higher difference above this value. At the end points, the stress difference for PP7B7G and PP5B5G lay at approximately 3%. 

The goal of the numerical study was to analyze the correlation between the experimentally gained data and the numerically calculated tensile strength of a specimen fabricated from different composite compositions. By making use of the MF module of Digimat, the material properties were estimated according to the mean-field Mori-Tanaka homogenization theory of the program [27]. In order to model this composite, the ‘multi-inclusion homogenization‘ option, with the ‘multi-step method’, was chosen. The best curve fit can be observed for PP10B10G, which was the template for the reverse engineering process. The error of the maximum stresses of PP7B7G and PP5B5G lays at approximately 3% (both curves). The values presented in Table 1 are one data set (Digimat material), which is an input to the Digmiat-RP module (see Figure 5). A parallel work path was the simulation of the injection process in Moldex3D. The simulation result is the generated fiber orientation inside the tensile test specimen (manufacturing data) (Figure 5, left hand side). During the injection process, the fiber directions near the gate are diversified. Within the slim section of the specimen, the fibers have nearly a uniform direction. As third input to the Digimat-RP, the structural model (Figure 5, right-hand side), was generated by making use of Ansys Mechanical. The model of the test specimen consists of 756 Solid186 elements and 4651 nodes. The right-hand face, marked with ‘B’ (purple marker), was set as the fixed support in all directions. A force was applied to the specimen (red arrow) at the opposite face. The maximum values were ramped up to a certain level during a time of one second, according to the measured forces at the tensile tests (see Table 3).

These data were handed over to Digimat-RP, which analyzed all the aforementioned input data, and calculated the tensile stress and strain of the force-loaded specimen. The results were imported to and visualized in Ansys.

**Figure 6:**
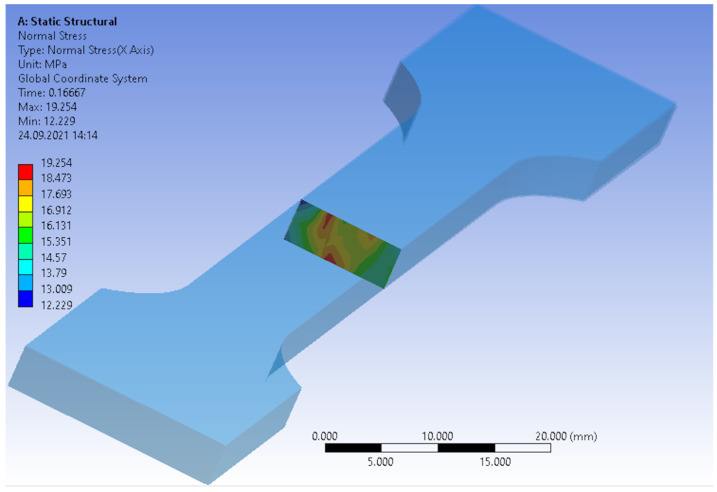
Cross-section of the specimen with visible stress distribution according to fiber orientation.

**Figure 7:**
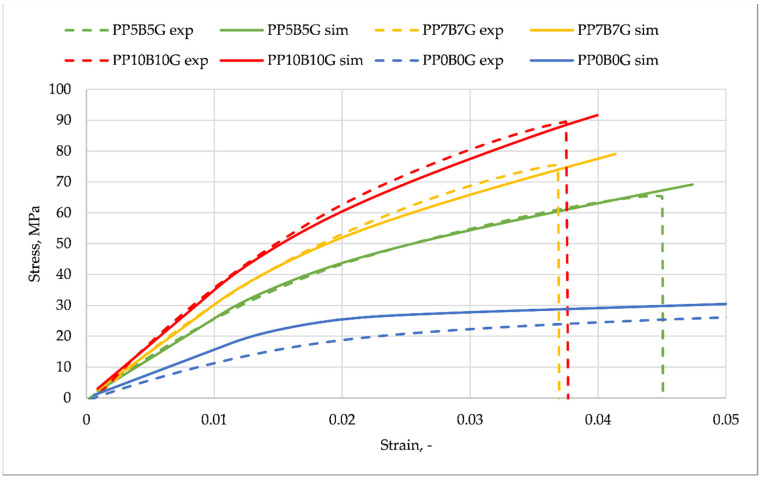
Stress–strain curves. Results of FEM analysis compared to measured data.

A numerical analysis of the tensile test of the multi-phase composite specimen was carried out. The input data were the boundary and load conditions, the fiber orientation and the homogenized material data of the polypropylene, and glass fiber and basalt fiber material. The computation was carried out with Digimat-RP, by making use of the Ansys solver. The cross-section area of the specimen is visible in Figure 6. In contrast to homogenous materials, an irregular stress distribution can be observed, due to the fiber orientation, generated by the manufacturing process. Figure 7 shows the comparison of the reverse engineered material of the FEM analysis (solid line), in comparison to the measurement data (dashed line) of the different material compositions. According to the added fiber distribution, the stresses are presented with different colors. The strain–stress curve, obtained by the Digimat-RP simulation, is based on the mean equivalent stress value of the cross-section and the maximum equivalent total strain of the specimen. 

The simulation values and experimental data correspond, to a high degree, within the elastic range of the composites. The error at the maximum stress of the composites lays at about 2% (1% for the template composite PP10B10G). For pure polypropylene, the values of each mass fraction (basalt and glass fibers) were set to the minimum value of 1 × 10^−6^. As result, it can be realized that the model does not give adequate results with such parameters. The error lays in a range of approximately 20%. A good correlation between the measurements and simulation was obtained with the enabled ‘large deflection’ option within the analysis setting. Additional simulations were carried out to validate what influence this option has on the results. The errors between the simulated and measured maximum stresses increased for PP10B10G, to 6%, to 4% for PP7B7G, and to 4% for PP5B5G. It can be assumed that the ‘large deflection’ option for the static structural calculation of mean-field homogenized materials is of high importance. In experimental results, the descent section represents the failure of the sample. Due to the fact that the simulation is calculated with an implicit solver, no failure of the material can be computed. Carrying out the model as explicit was not possible. By making use of FEM, the maximum stresses of the simulated parts with this material are not allowed to have higher values. 

To avoid numerical simulations with licensed tools that also require a lot of preparation, several models can be used to predict elastic modulus, such as the following: Voigt [48], Reuss [49], Tsai–Pagano [50], Hirsch [51], Halpin-Tsai [52], etc. In our study, basic models, such as Voight (E) and Reuss ($E_{⊥}$), were used, as follows:

(1) $$\{\begin{array}{l} E=E_{m}V_{m}+\overset{n}{\underset{i=1}{\sum }}E_{\mathrm{fi}}V_{\mathrm{fi}}\\ \frac{1}{E_{⊥}}=\frac{V_{m}}{E_{m}}+\overset{n}{\underset{i=1}{\sum }}\frac{V_{\mathrm{fi}}}{E_{i}} \end{array}$$

where E is the Young modulus of the composite, E_m_ is the elastic modulus of the matrix, E_f_ is the elastic modulus of the fibers, and V_f_ is the fiber volume fraction.

**Figure 8:**
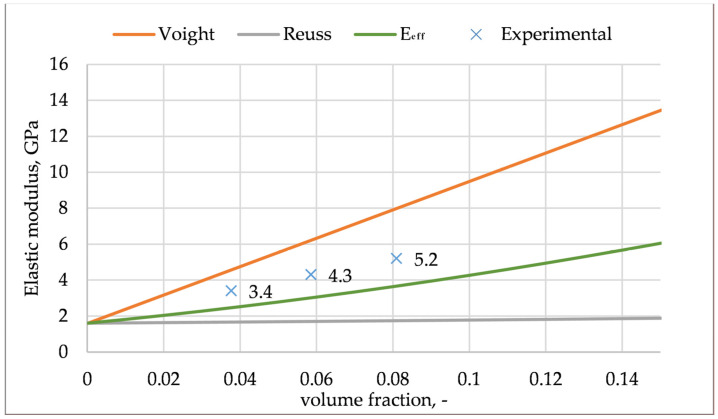
Comparison of modulus experimental results with Voight, Reuss method and method proposed by Wiśniewska (E_eff_).

Additionally, the new method, proposed by Wiśniewska, was also used to predict the elastic modulus [53,54]. In this method, the composite is described as a multi-phase material, and the inclusions are randomly distributed and oriented. Figure 8 compares the experimental results with all the proposed models. Iso-strain Voigt, which refers to parallel configurations, and iso-strain Reuss, can be used to calculate the upper and lower bounds for elastic modulus, respectively. The real elastic modulus should lay between these two bounds. The new method was more accurate; however, our experimental results have higher values than the calculated values. The mechanical properties of the material are a combination of the properties of the matrix and fillers, and the ability to transfer stresses at the interface between the fiber and the matrix. The matrix stiffness, and the fiber content, orientation, and stiffness have a major impact on the elastic modulus. In the injection molded composites, the fibers tend to mostly arrange along the flow direction. In the presented models, the orientation of fibers was not accurate.

The fatigue properties are useful information for materials used in long-term applications and subjected to cyclic loads or vibrations, especially for automotive applications. The production technology of polymer materials causes a heterogeneous state of stress, so the hysteresis loop fields changed in the successive cycles of loading and unloading. The analysis of the hysteresis loop allows the energy losses during loading and unloading the sample to be determined. The viscoelastic behavior of polymers means that, during deflection, the stresses and deformations are not in one phase, but a delay occurs, and, as a consequence, a mechanical hysteresis loop is created, which results in energy dissipation [55]. Polypropylene is a thermoplastic material with viscoelastic behavior. The addition of rigid basalt and glass fiber changed the behavior of the material. There are different sources that cause energy dissipation in composites. Failure of the composite can be initiated by cracks in the polymer matrix and poor adhesion of fillers to the matrix. External loads lead to local cracks, mainly at the boundaries of the matrix and the fibers. Also, test parameters, such as amplitude of stress or strain, can affect the results [56].

**Figure 9:**
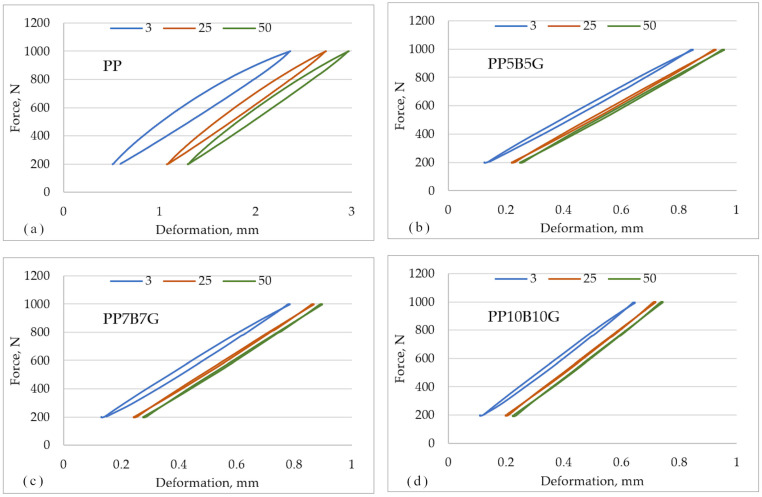
Hysteresis loops of the tested materials. (**a**) PP, (**b**) PP5B5G, (**c**) PP7B7G, (**d**) PP10B10G.

In Figure 9, the hysteresis loops for polypropylene and its composite are compared. To better visualize, only the 3rd, 25th, and 50th loops are presented. The largest loops and the highest deformation can be observed for polypropylene. With the subsequent cycles, the reduction in loop surface area is noticeable for all the materials.

**Figure 10:**
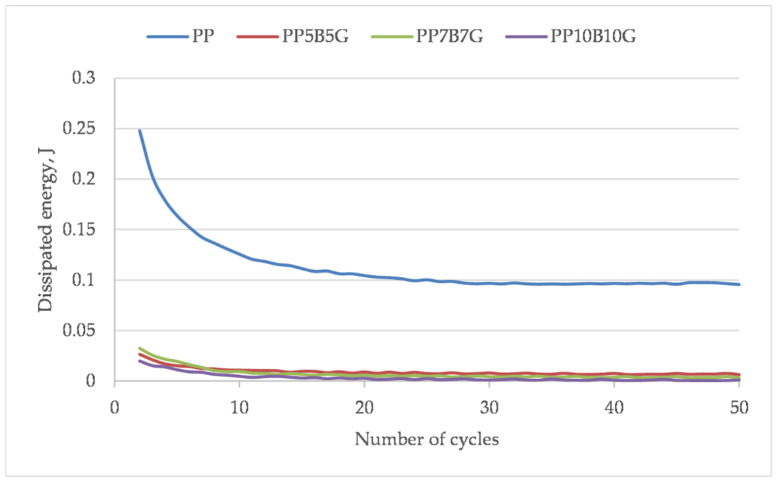
Dissipation energy of successive hysteresis loops of sample load and unload.

**Figure 11:**
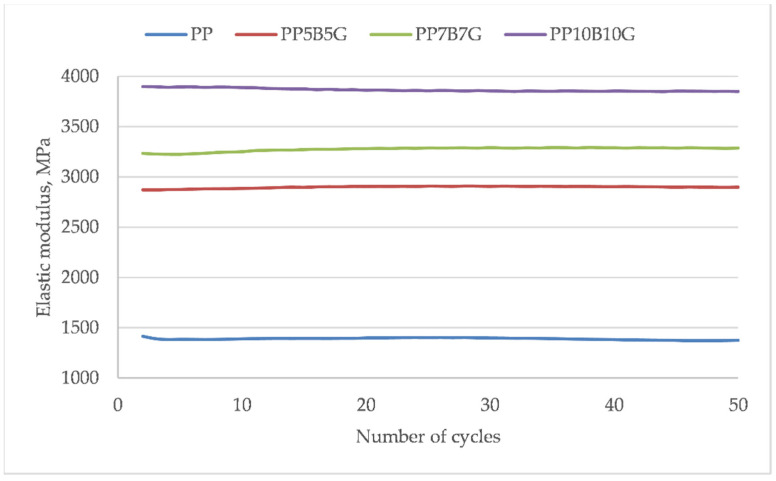
Elastic modulus of successive hysteresis loops of sample load and unload.

Based on the hysteresis loops, the dissipation energy was calculated (Figure 10). The addition of rigid basal and glass fibers reduced the toughness of the material; however, there is no significant difference between 10 and 20 wt.%. The fibers effectuated a lower material deformation and the strength and modulus increased. The change in dissipation energy in the subsequent loops shows a significant reduction in energy dissipation for the first few loops. Then, stabilization of the properties can be observed. Especially, the fiber-reinforced composites showed an earlier stabilization. This tendency was also described by Mazurkiewicz [57]. Within the micro-areas of bondage between the fibers and matrix (which are more or less randomly oriented), critical stresses can occur during the first load cycles. The first loops eliminate the local highly stressed areas within the material, during which a cracking of the adhesive connections between the fibers and the matrix occurs. Figure 11 shows the elastic modulus of each hysteresis loop of the given material. A linear trend of the elastic modulus, after the load–unload cycles, can be observed. The 50 cycles did not cause a significant change in the elastic modulus. 

**Figure 12:**
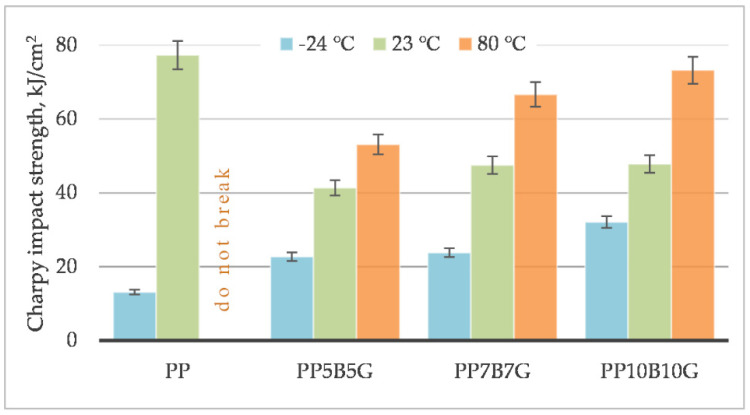
Unnotched Charpy impact strength of basalt/glass polypropylene composites at −24 °C, 23 °C and 80 °C.

Charpy impact tests were conducted, and the results are shown in Figure 12. Neat polypropylene has a significantly high impact strength. The impact strength decreased for composites reinforced by basalt and glass fibers. It decreased approximately twice; however, in a previous research study, the impact strength was lower for hybrid composites with basalt/carbon [58]. The higher fiber content increased the impact strength, although the values are lower than for neat polypropylene. The impact toughness of a composite depends both on the properties of the matrix and the fiber content, thus the adhesion between the fiber and the matrix, as well as the debonding and pull-out effect, are decisive. The temperature has a significant effect on the impact properties. The negative temperature caused a drop in strength impact (polymer became more brittle), and the elevated temperature caused an increase (polymer is more ductile, due to the intensive movement of chains).

**Figure 13:**
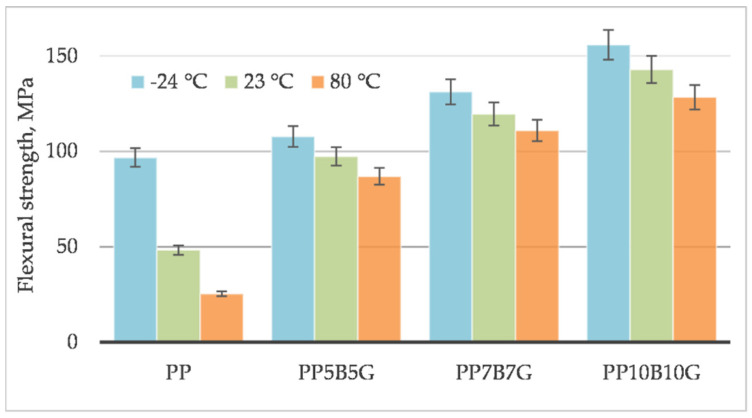
Flexural strength of basalt/glass polypropylene composites at −24 °C, 23 °C and 80 °C.

**Figure 14:**
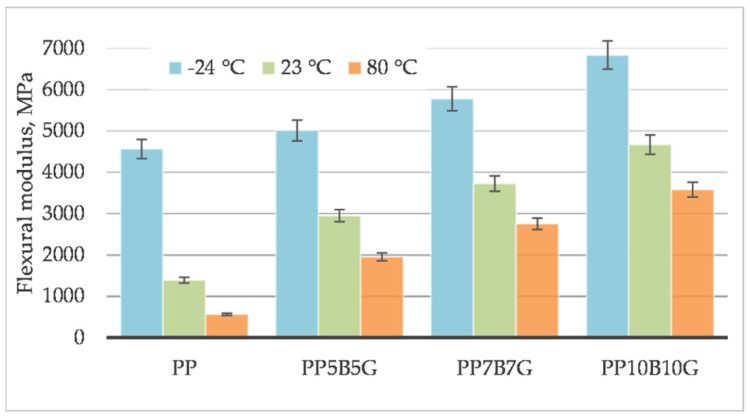
Flexural modulus of basalt/glass polypropylene composites at −24 °C, 23 °C and 80 °C.

Figure 13 and Figure 14 compare the mechanical properties evaluated in the flexural test. The flexural strength increased by 296% and the flexural modulus increased by 292% for a composition with 20%, by weight, of fibers. The composite climbed up to a value of 143 MPa, which was a higher value compared to the tensile strength of 88 MPa. The flexural strength values of plastics differ significantly from the tensile strength; the flexural strength of plastics is practically always greater than the tensile strength. Material strength is affected by randomly distributed defects in the material, which is described by the statistical Weibull distribution. During the bending test, only half of the sample is stressed, while, during the tensile test, the whole sample is stressed. Due to this, fewer defects are taken into account when bending. The moduli of elasticity are not subject to this regularity; as a rule, their values are quite similar (5185 MPa for a tensile modulus, and 4669 MPa for a flexural modulus).

### 3.2. Micrograph Observation

**Figure 15:**
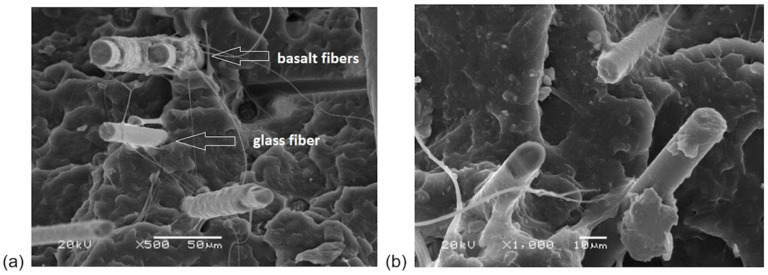
Micrographs of fracture surfaces of PP10B10G—magnification: (**a**) 500×, (**b**) 1000×.

Figure 15 presents the micrographs of the composite fracture surfaces after the tensile test for PP10B10G. Different sizes of fibers are noticeable; glass fiber has a diameter of 10 μm, and basalt fiber has a diameter of 17 μm. The fibers are well embedded into the polymer matrix. Good adhesion of the matrix to the fiber is noticeable, especially for the basalt fibers, where the matrix encloses them. The addition of a coupling agent helped to create a strong bond between the fibers and the matrix. Good fiber distribution and adhesion to the polymer matrix had a positive effect on the mechanical properties. 

The length of the glass fibers was 6 mm, and of the basalt fibers, it was 6.4 mm. On the one hand, in molded parts, a short fiber length, starting at 0.1 mm, is preferred, to ensure a good production process, but, on the other hand, the mechanical properties of fiber-reinforced composites improve with increasing fiber length [59,60]. This fiber length was used to maintain good processability and to obtain good mechanical properties. However, during injection molding, a fiber’s damage occurs because of high shear mixing conditions.

## 4. Conclusions

It can be concluded that basalt and glass fibers can be mixed together to produce composites by injection molding, which are based on polypropylene. A good fiber–matrix interface was achieved by adding coupling agents. This had a positive impact on the mechanical properties of the materials. The tensile strength increased by 306% and the tensile modulus by 333% for a composition with 20%, by weight, of fibers. The usage of components with these composites is assured in a wide temperature range. At cyclic uniaxial loading and unloading of the specimen, no significant decrease in the elastic modulus could be observed. Because of the high strength-to-weight ratio and anticorrosion properties of these advanced polymer hybrid composites, they can be perfectly used for applications within the automotive, aerospace, and sporting industries. In general, basalt/glass fiber polypropylene hybrid composites can be used as a substitution material, instead of pure glass fiber-reinforced plastics, with the ecological aspect in the background because of its natural origin. Keeping in mind that the maximum addition of fibers is up to 20% by weight, this material can be used for lightweight applications. Due to the fact that building prototypes is time consuming and expensive, numerical simulations are carried out in the first step. By making use of the methodology of reverse engineering and different software programs, the estimated material properties can be utilized to simulate the strength of new components, built up of these composites. With a relatively low difference in maximum stress of approximately 2%, the simulation data can be used for future FEA.

## Figures and Tables

**Table 1:** Estimated Digimat-MF material parameters.

Properties	Polypropylene	Glass Fiber	Basalt Fiber
Constitutive law	Elastoplastic	Elastic	Elastic
Elasticity	Isotropic	Isotropic	Isotropic
Density (kg/mm^3^)	9.2 × 10^−7^	2.5 × 10^−6^	2.6 × 10^−6^
Young’s modulus (MPa)	1600	75,000	89,000
Poisson’s ratio (-)	0.42	0.22	0.25
Plasticity model	J2	-	-
Isotropic hardening model	Hardening modulus (MPa): 9.5 Hardening exponent (-): 300 Linear hardening modulus (MPa): 4	-	-
Kinematic hardening model	Linear hardening modulus (MPa): 120 Restoration parameter (-): 50	-	-

**Table 2:** The density and composition content of composites.

Symbol	Composition	Density, g/cm^3^
PP	neat polypropylene HP 500N	0.886 ± 0.001
PP5B5G	PP + 5 wt.% basalt fibers + 5 wt.% glass fibers + 3 wt.% MAPP	0.974 ± 0.003
PP7B7G	PP + 7.5 wt.% basalt fibers + 7.5 wt.% glass fibers + 3 wt.% MAPP	0.995 ± 0.005
PP10B10G	PP + 10 wt.% basalt fibers + 10 wt.% glass fibers + 3 wt.% MAPP	1.017 ± 0.001

**Table 3:** Maximum tensile force within FEA.

Material	PP	PP5B5G	PP7B7G	PP10B10G
Maximum force (N)	1200	2700	3100	3600

## Data Availability

The data presented in this study are available on request from the corresponding author.
